# Characterization and Functional Implications of the Nonexpressor of Pathogenesis-Related Genes 1 (*NPR1*) in *Saccharum*

**DOI:** 10.3390/ijms23147984

**Published:** 2022-07-20

**Authors:** Shoujian Zang, Liqian Qin, Zhennan Zhao, Jing Zhang, Wenhui Zou, Dongjiao Wang, Aoyin Feng, Shaolin Yang, Youxiong Que, Yachun Su

**Affiliations:** 1 Key Laboratory of Sugarcane Biology and Genetic Breeding, Ministry of Agriculture and Rural Affairs, Fujian Agriculture and Forestry University, Fuzhou 350002, China; zangshoujian2020@163.com (S.Z.); qinliqian0000@163.com (L.Q.); zn2008@139.com (Z.Z.); zhashoveljing@163.com (J.Z.); zwh19961546644@126.com (W.Z.); dongjiaow@126.com (D.W.); feng_aoyin98@163.com (A.F.); cbttxsysl@foxmail.com (S.Y.); 2 Yunnan Key Laboratory of Sugarcane Genetic Improvement, Sugarcane Research Institute, Yunnan Academy of Agricultural Sciences, Kaiyuan 661600, China; 3 Key Laboratory of Genetics, Breeding and Multiple Utilization of Crops, Ministry of Education, College of Agriculture, Fujian Agriculture and Forestry University, Fuzhou 350002, China

**Keywords:** sugarcane, *NPR1*-like gene, genome-wide analysis, expression profile, defense response

## Abstract

Sugarcane (*Saccharum* spp.) is an important sugar and energy crop worldwide. As a core regulator of the salicylic acid (SA) signaling pathway, nonexpressor of pathogenesis-related genes 1 (*NPR1*) plays a significant role in the response of the plant to biotic and abiotic stresses. However, there is currently no report on the *NPR1*-like gene family in sugarcane. In this study, a total of 18 *NPR1*-like genes were identified in *Saccharum spontaneum* and classified into three clades (clade I, II, and III). The *cis-*elements predicted in the promotors revealed that the sugarcane *NPR1*-like genes may be involved in various phytohormones and stress responses. RNA sequencing and quantitative real-time PCR analysis demonstrated that *NPR1*-like genes were differentially expressed in sugarcane tissues and under *Sporisorium scitamineum* stress. In addition, a novel *ShNPR1* gene from *Saccharum* spp. hybrid ROC22 was isolated by homologous cloning and validated to be a nuclear-localized clade II member. The *ShNPR1* gene was constitutively expressed in all the sugarcane tissues, with the highest expression level in the leaf and the lowest in the bud. The expression level of *ShNPR1* was decreased by the plant hormones salicylic acid (SA) and abscisic acid (ABA). Additionally, the transient expression showed that the *ShNPR1* gene plays a positive role in *Nicotiana benthamiana* plants’ defense response to *Ralstonia solanacearum* and *Fusarium solani* var. *coeruleum*. This study provided comprehensive information for the *NPR1*-like family in sugarcane, which should be helpful for functional characterization of sugarcane *NPR1*-like genes in the future.

## 1. Introduction

The nonexpressor of pathogenesis-related genes 1, also known as noninducible immunity protein 1, salicylic acid (SA) insensitive 1, or pathogenesis-related (PR) gene, is essential for the plant response to pathogen challenge [1,2,3]. NPR1 plays a significant role in the establishments of systemic acquired resistance (SAR) and induced systemic resistance (ISR) [4]. The *NPR1* gene was first discovered from the *Arabidopsis thaliana npr1* mutant in the research on the plant SAR signaling pathway [1,5]. Previous studies have shown that in the *npr1* mutant, the gene encoding the PR protein was not expressed, and the SAR reaction cannot be activated, thereby failing to develop disease resistance [1,2,5]. As a key regulator of SA signaling transduction in plants, NPR1 is located downstream of the SA signaling pathway and upstream of the PR protein gene. NPR1 is the intersection of multiple resistance signaling pathways and plays an important role in regulating the overall disease resistance of plants [4,6].

At present, two to six *NPR1*-like genes have been found in the plant species. In *Arabidopsis* alone, five additional *NPR1*-like genes (*AtNPR2*, *AtNPR3*, *AtNPR4*, *AtNPR5/AtBOP1*, and *AtNPR6/AtBOP2*) have been described [7,8,9,10]. The encoding proteins for these *NPR1*-like genes contain two well-documented domains, including ankyrin repeats and BTB/POZ (broad complex, tramtrack, bric a brac/pox virus, and zinc finger) [5,8,11,12]. Phylogenetic analysis revealed that the *NPR1*-like gene family can be classified into three clades [13,14]. As reported, each clade of the *NPR1*-like gene family appears to have its own set of functional requirements [5,9,15]. Clade I members (AtNPR1 and AtNPR2) are involved with positive SAR regulation and clade II members (AtNPR3 and AtNPR4) are negatively involved in SAR regulation [5,15], while clade III members (AtBOP1 and AtBOP2) are related to the growth and development of plant leaves and flowers [8]. Most of the peptides encoded by the *PR* genes are effectors directly involved in the defense against pathogens [16]. Once infected by pathogens, the accumulation of SA in plants can cause changes in the redox state of plant cells, resulting in the cleavage of disulfide bonds between NPR1 oligomer molecules in the cytoplasm into monomers and their transfer to the nucleus [17]. NPR1 is phosphorylated upon entry into the nucleus and is subsequently degraded by the 26S proteasome after cullin 3-mediated ubiquitination [12]. Only the phosphorylated NPR1 can activate SAR [18]. The key role of the *NPR1* gene in plant disease resistance, especially in the establishment of SAR, has been confirmed by much research [6,19]. Overexpression of the *NPR1* gene in *Oryza sativa* [20], *Citrus reticulata* Blanco [21], *BraSsica campestris* [22], *GoSsypium* spp [23], *Vitis vinifera* [24], and *Triticum aestivum* [25] can effectively improve plant resistance to rice sheath blight, ulcer disease, *Pseudomonas syringae*, cotton root rot, powdery mildew, and gibberellic disease. Thus, NPR1, as the main regulator of plant defense signals, plays a significant role in a wide range of defense responses [6].

Sugarcane (*Saccharum* spp.), which belongs to monocotyledonous plants of the Gramineae genus *Saccharum*, is an important global sugar and biofuel feedstock crop in more than 110 tropical and subtropical countries [26]. As with other crops, sugarcane production is affected by a variety of adverse environmental conditions, including drought, cold, and disease, especially fungus [26,27]. Screening sugarcane genes in response to adversity stress will provide a basis for the cultivation of excellent sugarcane varieties with stress resistance [26]. Therefore, the use of biotechnology and genetic engineering technology can speed up the process of sugarcane breeding and improve the quality of sugarcane varieties. It has been confirmed that various plant species overexpressing *NPR1* or its orthologs can enhance disease resistance to several kinds of pathogens [24,28,29]. However, a systematic investigation of the evolution and biological function of the *NPR1-*like genes in *Saccharum* has not yet been reported. In the present study, 18 *SsNPR* genes were identified from the genome of *S*. *spontaneum* [30]. Their classification, gene structure, motif composition, chromosomal distribution, evolution, *cis*-regulatory elements (CREs), and synteny analysis were performed. In addition, the expression profiles of *NPR1*-like genes in different sugarcane tissues and under *S*. *scitamineum* stress were measured using RNA sequencing (RNA-seq) and quantitative real-time PCR (qRT-PCR). Furthermore, a clade II *NPR1*-like gene, *ShNPR1*, was successfully cloned from sugarcane variety ROC22. The sequence characteristics, subcellular localization, tissue-specific expression, gene expression patterns under different stresses, and transient expression of *ShNPR1* in *Nicotiana benthamiana* after inoculation with the tobacco pathogens *Ralstonia solanacearum* and *Fusarium solani* var. *coeruleum* were analyzed. This study provides a reference for systematically understanding the characteristics of the sugarcane *NPR1*-like gene family and the identification of its function in disease resistance.

## 2. Results

### 2.1. Identification and Characterization of the NPR1-like Gene Family

A total of 18 *NPR1-*like gene sequences were identified in the *S. spontaneum* genome. These genes that belong to alleles were designated as the same name followed by the letters “a”, “b”, “c”, and “d”, and duplicated genes were designated as the same name followed by the letters “e”, “f”, and “g” [30] (Table S1). Among these 18 *SsNPR* genes (*SsNPR1*–*5*), *SsNPR2* and *SsNPR3* had four alleles (*SsNPR2a*/*2b*/*2c*/*2d* and *SsNPR3a*/*3b*/*3c*/*3d*), and *SsNPR1* and *SsNPR4* had two alleles (*SsNPR1a*/*1b* and *SsNPR4a*/*4b*). Additionally, *SsNPR5e*, *SsNPR5f*, and *SsNPR5g* all originated from dispersed duplications. The encoded SsNPR proteins were 429-647 amino acid (aa) residues in length, with the molecular weight (MW) ranging from 45.42 kDa to 70.57 kDa. Their isoelectric points (*p*Is) varied from 5.48 to 6.68, and the instability index was all greater than 40. In addition, all these SsNPRs were predicted to be acidic proteins and located in the nucleus (Table S1). The secondary structure of the 18 SsNPR proteins was mainly composed of an α-helix (43.98–56.57%), irregular curl (32.87–43.39%), and extended chain (5.75–9.19%), but lacked a β-sheet (Table S2).

### 2.2. Phylogenetic Analysis of the NPR1-like Gene Family

**Figure 1:**
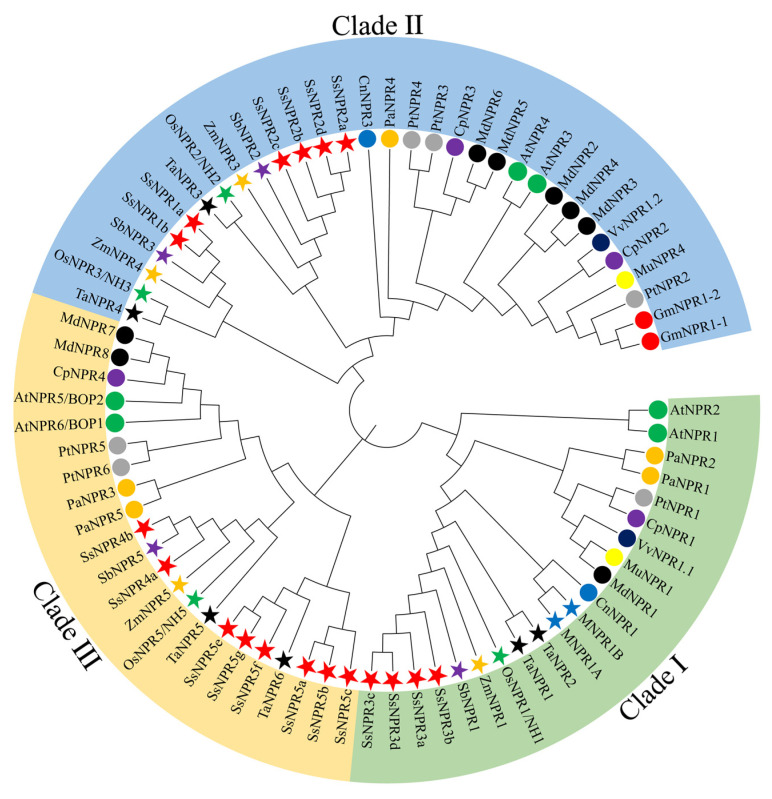
Phylogenetic analysis of NPR1 homologous proteins from 15 plant species. The MEGA X with the neighbor-joining method was used to conduct the phylogenetic tree. NPR1-related proteins came from five monocot species (*Musa acuminate* (M), *Oryza sativa* (Os), *Sorghum bicolor* (Sb), *Triticum aestivum* (Ta), and *Zea mays* (Zm)) and nine dicot species (*Arabidopsis thaliana* (At), *Carica papaya* (Cp), *Cocos nucifera* (Cn), *Glycine max* (Gm), *Malus domestica* (Md), *Morus multicaulis* (Mm), *Persea americana* (Pa), *Populus trichocarpa* (Pt), and *Vitis vinifera* (Vv)). All the GenBank accession numbers of the NPR1 homologous proteins are listed in Table S3. Three major clades are distinguished with three colors, and the NPR1 homologous proteins from different plant species are indicated by different colors of circles and stars.

To explore the evolutionary relationship of the *SsNPR1*-like gene family, NPR1-like protein sequences from five monocots and nine dicots were used to construct the phylogenetic tree (Table S3). Figure 1 showed that these NPR1-like proteins were divided into three clades, including clade I, II, and III. SsNPR3a/3b/3c/3d were classified into clade I, including AtNPR1 and AtNPR2, which were reported as positive regulators of SAR [15]. SsNPR1a/1b and SsNPR2a/2b/2c/2d were classified into clade II, among which AtNPR3 and AtNPR4 served as negative regulators of SAR [10]. The other eight SsNPR proteins (SsNPR4a/4b and SsNPR5a/5b/5c/5e/5f/5g) were classified into clade III, along with AtNPR5/AtBOP1 and AtNPR5/AtBOP2, which participated in the organ determinacy and symmetry [8,31]. The NPRs of monocotyledonous and dicotyledonous plants were not clustered separately, indicating that the NPR sequences were conserved among plant species.

### 2.3. Gene Structures and Conserved Motifs of the NPR1-like Gene Family

**Figure 2:**
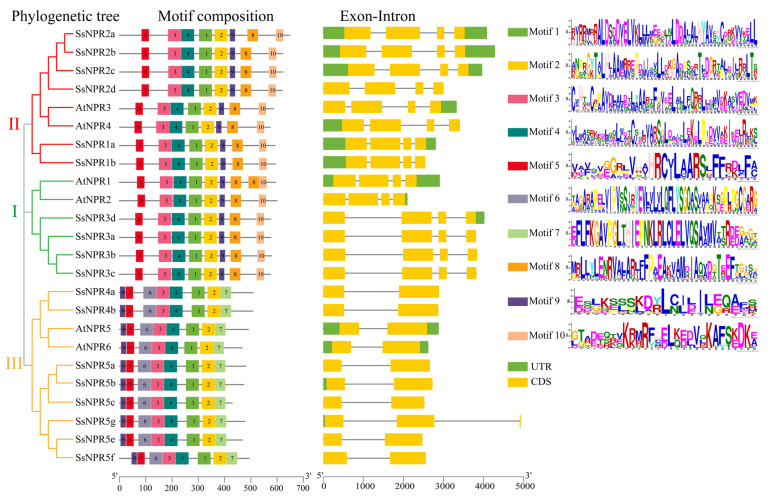
Phylogenetic tree, conserved motif, and gene structure of the NPR1-like proteins in *Saccharum spontaneum* and *Arabidopsis.* Different colors on the phylogenetic tree represent different clades of the *NPR1*-like gene family and different colored boxes indicate different motifs (motif 1–10). The noncoding sequences, exons, and introns are shown as green boxes, yellow boxes, and black lines in the exon–intron structures, respectively. The length of proteins and genes is estimated using the scale at the bottom.

To further understand the potential functions of *SsNPR1*-like genes, their conserved motifs and gene structures were analyzed. A total of 10 different conserved motifs were observed in the 18 NPR1-like proteins (Figure 2 and Table S4). NPR1-like members that belonged to the same clade had similar types, arrangements, and number of motifs. All these NPR1-like proteins contained motifs 1, 2, 3, 4, and 5. Interestingly, there were eight types of motifs in all three clades, but their motif composition was not the same. For example, motifs 6 and 7 were absent in clades I and II, while motifs 8 and 10 were not contained in clade III. Furthermore, the exon–intron organizations showed that the exon numbers were from two to four in the NPR1-like proteins. As expected, most of the *NPR1*-like genes in the same clade had similar patterns of exon–intron distributions and positions. These *NPR1*-like genes in clades I and II had four exons. Six *NPR1*-like genes in clade III (*SsNPR4a/4b* and *SsNPR5a/5c/5e/5f*) presented similar exon–intron structures. Most members of the *NPR1*-like genes in different clades had similar gene structures, indicating that *NPR1*-like sequences were conserved in evolution.

### 2.4. Chromosomal Location, Gene Duplications, and Synteny Analysis of the NPR1-like Gene Family

**Figure 3:**
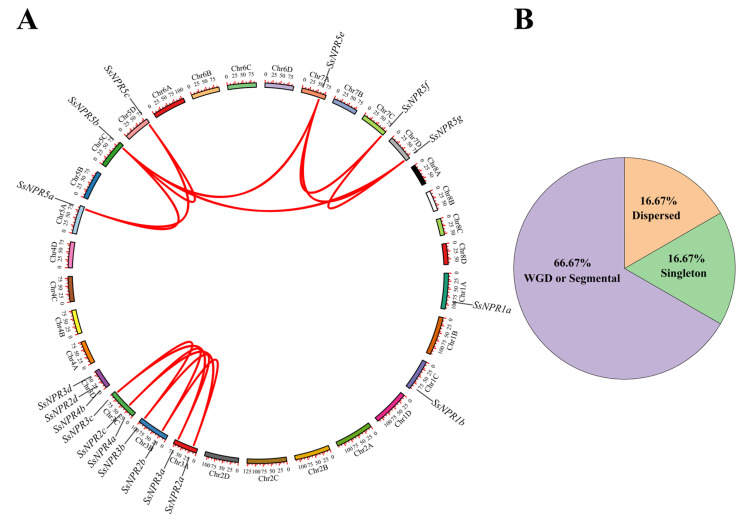
Evolutionary history of *NPR1*-like genes in *Saccharum spontaneum*. (**A**) Synteny analysis of the *NPR1*-like genes in *S. spontaneum*. The red lines represent the *SsNPR1*-likes that have been replicated. (**B**) The distribution of gene duplications among *SsNPR* genes. WGD represents the whole-genome duplication.

As illustrated in Figure 3, the distribution of 18 *SsNPR1*-like genes on the 12 *S. spontaneum* chromosomes was uneven. Chromosome 3D contained three *SsNPR1*-like genes, and Chromosomes 3A, 3B, and 3C each contained two *SsNPR1*-like genes. The remaining eight chromosomes each contained one *SsNPR1*-like gene (Figure 3A). A total of 14 segmental duplication gene pairs in 12 *SsNPR1*-likes were observed, of which 11 pairs (78.57%) occurred between alleles and three pairs (21.43%) occurred between nonalleles (Figure 3A and Table S5). The nonsynonymous (Ka) and synonymous (Ks) nucleotide substitution rates and the Ka/Ks of these *SsNPR1*-like gene pairs were calculated (Table S5). The results showed that among the 13 of 14 *SsNPR1*-likes, Ka/Ks was <1, and the remaining one *SsNPR1*-like gene pair (*SsNPR2b*/*SsNPR2c*) did not have a value. Therefore, purifying selection might be the primary pressure that preserves the function of *SsNPR1*-likes. Furthermore, to explore the expansion mechanisms of the *NPR1*-like genes, the whole-genome duplication (WGD)/segmental, and dispersed, proximal and tandem events were analyzed using chromosomal information of *S*. *spontaneum*. The result showed that 12 WGD/fragment repetitive genes (66.67%), three scattered repetitive genes (16.67%), and three single copies (16.67%) were detected in the 18 sugarcane *NPR1*-like genes (Figure 3B and Table S6).

### 2.5. Promoter Analysis of the NPR1-like Genes

**Figure 4:**
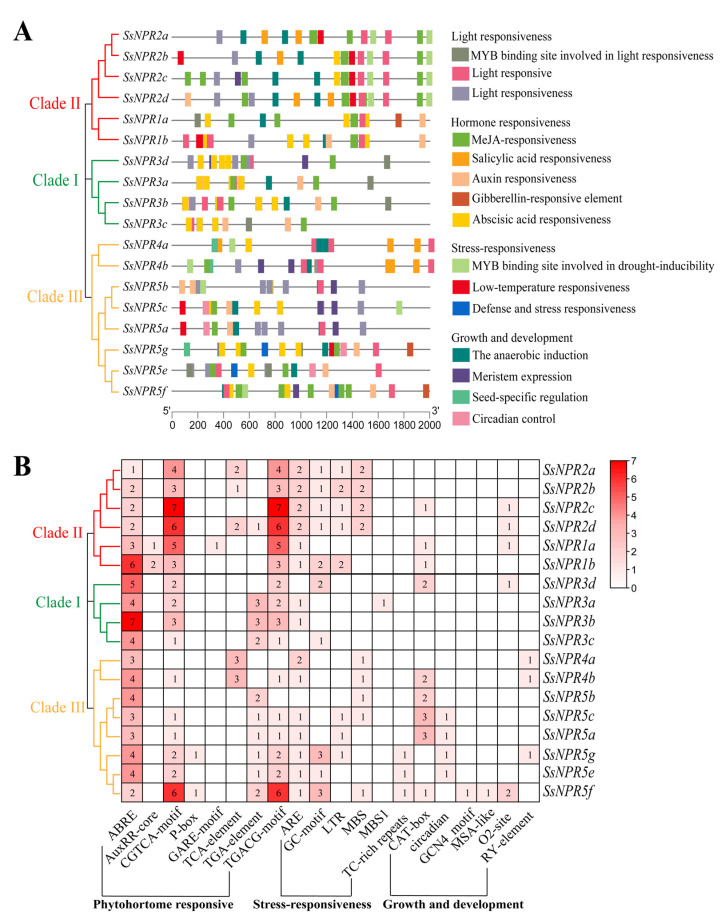
*Cis*-regulatory element (CRE) analysis of *NPR1*-like gene family. (**A**) The location distribution of different kinds of CREs in the promoter region. Different colored boxes correspond to different kinds of CREs and some CREs may overlap with other CREs. (**B**) The number of CREs in the promoter region of the *NPR1*-like genes. The number of each CRE is shown in the heatmap box, and blank indicates that there was no corresponding CRE. Different colors on the phylogenetic tree represent different groups of the *NPR1*-like gene family.

The promoter regions of the *SsNPR1*-like genes from *S. spontaneum* were submitted to the PlantCARE database to search the *cis-*elements. Four types of CREs, which were related to light, plant growth/development, hormone, and stress-related responses, were predicted in the 2000-bp promoters of the 18 *NPR1*-like genes (Figure 4, Tables S7 and S8). Promoters of the 18 *NPR1*-like genes all contained light response elements. The abscisic acid (ABA) and methyl jasmonate (MeJA) response elements existed in the promoters of 100% and 88.89% *NPR1*-like genes. In addition, the promoters of twelve (66.67%), five (27.78%), and three (16.67%) *NPR1*-like genes contained auxin, SA response, and gibberellin (GA) elements, respectively (Table S8). Elements related to the stress response, such as anoxic-induction elements (AREs) or GC-motifs (enhance anoxic specific inducibility), MYB binding sites, which are involved in drought-inducibility (MBSs), low-temperature-responsive elements (LTRs), and defense and stress response elements (TC-rich repeats), were found in the promoters of ten (55.56%), eight (44.44%), eight (44.44%), and three (16.67%) *NPR1*-like genes, respectively. The *SsNPR3a* promoter contained a MYB binding site, while the *SsNPR5g/5e/5f* promoters contained a defense and stress-responsive element. Regarding plant growth and development, promoters of nine (50%) *NPR1*-like genes contained a CAT-box, which is a *cis-*acting regulatory element related to meristem expression. Promoters of five (27.78%) and four (22.22%) *NPR1*-like genes may have been involved in zein metabolism regulation and circadian control. Promoters of three (16.67%) *NPR1*-like genes (*SsNPR4a/4b/5g*) contained an element of seed-specific regulation. These results indicate that the *NPR1*-like genes may be widely involved in the response to various stressors and in the regulation of plant growth and development.

### 2.6. Tissue-Specific Expression of NPR1-like Genes and Their Expression Profiles during the Interaction between Sugarcane and Smut Pathogen

**Figure 5:**
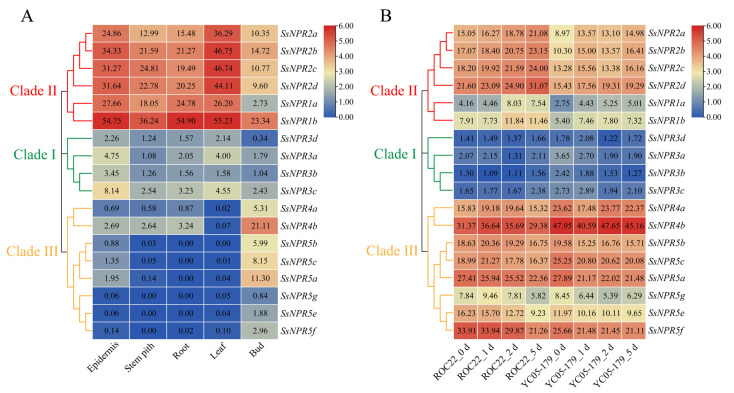
Expression profile of *NPR1*-like genes in various tissues of sugarcane and under smut pathogen stress. (**A**) Expression profile of *NPR1*-like genes in different tissues of sugarcane hybrid ROC22. (**B**) Expression profile of *NPR1*-like genes in the interaction between different sugarcane genotypes and *Sporisorium scitamineum*. ROC22_0 d/1 d/2 d/5 d represents the sugarcane smut-susceptible variety ROC22 under *S*. *scitamineum* treatment for 0 d, 1 d, 2 d, and 5 d, respectively. YC05–179_0 d/1 d/2 d/5 d represents the sugarcane smut-resistant variety YC05–179 under *S*. *scitamineum* treatment for 0 d, 1 d, 2 d, and 5 d, respectively. The clustering tree on the left side of the figure is constructed using MEGA X with the neighbor method, and different colors on the phylogenetic tree represent different NPR1-like clades. The expression heatmap of *NPR1*-like genes is constructed by TBtools with the transcript level transformed by log2 (FPKM). The FPKM is the number of fragments per kilobase of transcript per million mapped, and its value is displayed in each heatmap box.

Among the 18 *NPR1*-like genes, 12 showed expression (FPKM > 0) in all sugarcane tissues based on the transcriptome data. The number of *NPR1*-like genes expressed (FPKM > 0) in the sugarcane epidermis, stem pith, root, leaf, and bud was 18, 15, 13, 17, and 18, respectively. In clade II, the expression level of *SsNPR1b* in the five ROC22 tissues was higher than that of the other *SsNPR1*-likes, indicating that this gene may play an important role in sugarcane growth and development (Figure 5A). *SsNPR2a/2b/2c/2d* exhibited the highest transcript abundance in the leaf than those in the other tissues. In clade I, the expression level of *NPR1*-like genes in the epidermis was the highest. However, the expression level of all members in clade I was lower than that in clade II. In clade III, the expression level of *SsNPR5a/5b/5c/5e/5f/5g* exhibited low or undetectable expressions (FPKM < 1) in the stem pith, root, and leaf, while the expression level of all genes was the highest in the bud.

The expression pattern of *NPR1*-like genes during the interaction between sugarcane smut-susceptible variety ROC22/-resistant variety YC05–179 and smut pathogen for 0-5 d was analyzed (Figure 5B). The *SsNPR3a/3b/3c/3d* genes in clade II exhibited a low expression level (FPKM < 4) under the *S. scitamineum* stress. The expression level of clade II genes showed an upward trend, while the expression of *SsNPR4a/4b* and *SsNPR5a/5b/5c/5e/5f/5g* in clade III had a downward trend. It is worth noting that the expression level of clade II genes in the susceptible variety was higher than that in the resistant one, indicating that the regulatory pattern of the genes in clade II may be negative in the interaction between sugarcane and smut pathogen. In addition, in clade III, the expression level of *SsNPR5e/5f* in the resistant variety was lower than that in the susceptible one, while the expression trend of *SsMYC4b* was the opposite. The results indicate that all these *SsNPR1*-likes can be induced by the smut pathogen infection, and their expression patterns in the infected sugarcane-resistant and -susceptible varieties were different.

### 2.7. Cloning and Sequence Analysis of the ShNPR1 Gene

The expression of clade II gene members increased with time during the interaction between sugarcane smut susceptible variety ROC22/resistant variety YC05-179 and smut pathogen, suggesting that members of clade II respond positively to smut pathogen infection. Furthermore, the cDNA sequence of the homologous gene of *SsNPR2b* belonging to clade II gene members was successfully cloned from the sugarcane hybrid ROC22 leaves and termed *ShNPR1* (GenBank accession no. ON737832). The ORF length of *ShNPR1* was 1866 bp and encoded 621 amino acids, which contained 16 cysteine residues. The amino acid sequence similarity between ShNPR1 and SsNPR2b was as high as 99.80% (Table S9). The ShNPR1 protein contained a nuclear localization signal at the C-terminus, an N-terminal BTB/POZ domain, a NPR1-like C-terminal region, and ankyrin repeats in the central region (Figure S1).

**Figure 6:**
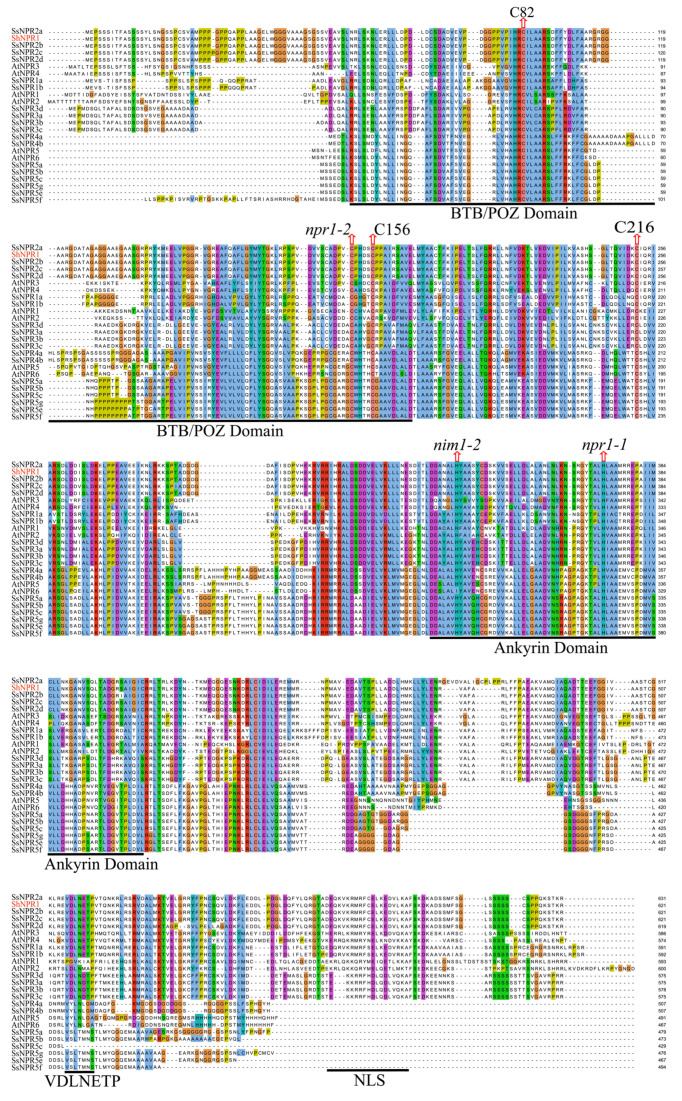
Multiple alignments of amino acid sequences of NPR1-like proteins in *Saccharum* and *Arabidopsis thaliana*. BTB/POZ, ankyrin conserved domains, EAR-like repression motif (VDLNETP), and nuclear localization signal (NLS) are underlined in black. npr1-1, npr1-2, and nim1-2 in AtNPR1 mutation sites, and the highly conserved cysteines residues (C82, C216, and C156) in AtNPR1 are shown by red arrows.

Multiple sequence alignments were performed on ShNPR1, SsNPR1-likes, and AtNPR1-likes (AtNPR1 to AtNPR6) with known functions to check the conservatism of residues, domains, and motifs (Figure 6). We found that npr1-2 (Cys150Tyr), nim1-2 (His300Tyr), and npr1-1 (His334Tyr) functional sites in AtNPR1 were completely conserved in all 25 NPR1-like proteins. C82, C216, and C156 cysteine residues in AtNPR1, which participated in its oligomer-monomer transition [19], were also highly conservative in NPR1-like proteins. There was a BTB/POZ domain in the N-terminal and an ankyrin conserved domain in the middle of the *Saccharum* and *Arabidopsis* NPR1-like proteins. In addition, NPR1-like proteins belonging to clades I and II have a nuclear localization signal (NLS) at the C-terminus.

### 2.8. ShNPR1 Was a Nuclear Localized Protein

**Figure 7:**
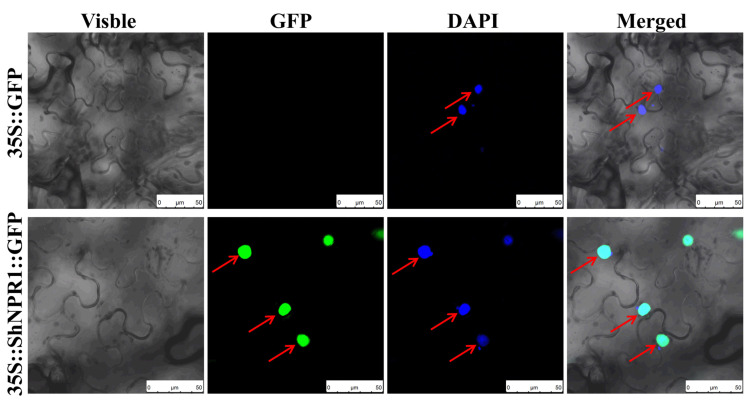
Subcellular location of ShNPR1 protein in *Nicotiana benthamiana* lower epidermal cells. Images of epidermal cells were captured using visible light, green fluorescence, blue fluorescence, and merged light. 35S::GFP, the *Agrobacterium tumefaciens* strain carried the empty vector pFAST-R05; 35S::ShNPR1::GFP, the *A*. *tumefaciens* strain carried the recombinant vector pFAST-R05-*ShNPR1*; pFAST-R05 expresses a gene fused to a green fluorescent protein gene (*GFP*), which followed the *ccdB* gene (with a stop codon); red arrows indicate nucleus. DAPI, 4′, 6-diamidino-2-phenylindole. Bar = 50 µm.

To understand the subcellular localization of the ShNPR1 protein, ShNPR1::GFP fusion proteins were expressed transiently in *N. benthamiana* leaf cells. In the control group, which contained an empty vector pFAST-R05 (35S::GFP) (the green fluorescent protein gene followed the *ccdB* gene, which contained a stop codon), no fluorescence was observed after *Agrobacterium*-mediated transformation and transient expression in *N. benthamiana* leaves (Figure 7). However, the fluorescence was observed in the nucleus of the lower epidermal cells of the 35S::ShNPR1::GFP leaves. Based on the Plant-mPloc predicted program, this result was in accordance with that in sequence analysis, which suggests that ShNPR1 was mainly localized to the nucleus. These findings prove that ShNPR1 is a nuclear localized protein.

### 2.9. ShNPR1 Was Constitutively Expressed in Sugarcane Tissues

**Figure 8:**
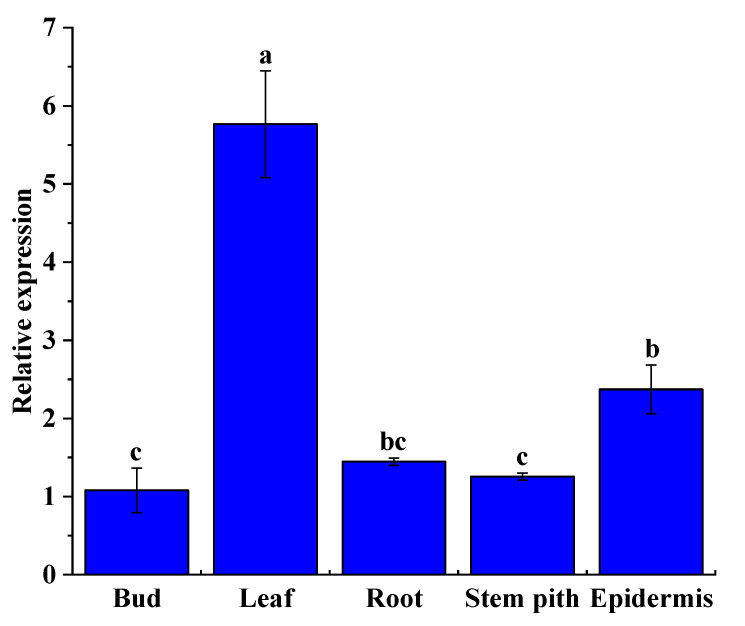
Tissue-specific expression analysis of *ShNPR1* in 10-month-old sugarcane ROC22 plants by qRT-PCR analysis. The expression level of glyceraldehyde-3-phosphate dehydrogenase (*GAPDH*) was used for data normalization. The bars represent the mean ± standard error (*n* = 3). Different lowercase letters on the top of the bars indicate the significant differences determined by Duncan’s new multiple range test (*p*-value < 0.05).

The relative expression level of the *ShNPR1* gene was detected in the 10-month-old ROC22 root, bud, leaf, stem pith, and stem epidermis by qRT-PCR (Figure 8). The results indicated that this gene was constitutively expressed in all these tissues. The lowest expression level of *ShNPR1* occurred in the bud, while its relative expression level in the leaf and epidermis was 5.34- and 2.20-fold higher than that in the bud, respectively.

### 2.10. Sporisorium Scitamineum, ABA, SA, and MeJA Triggered ShNPR1 Activation

**Figure 9:**
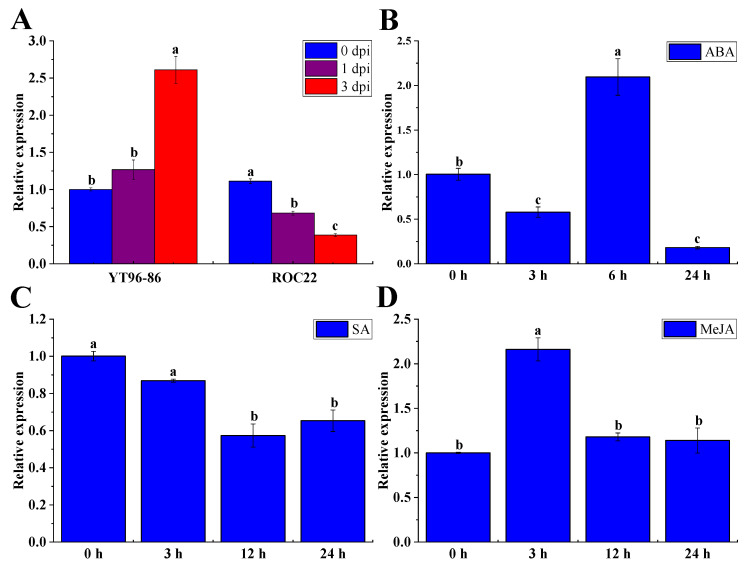
Expression patterns of *ShNPR1* in different stress conditions by qRT-PCR analysis. (**A**) Relative expression of *ShNPR1* in sugarcane after inoculation with *Sporisorium scitamineum*. YT96-86 was a sugarcane smut-resistant variety. ROC22 was a sugarcane smut-susceptible variety. dpi: days post-inoculation. (**B**–**D**) Relative expression of *ShNPR1* in 4-month-old ROC22 plantlets under abscisic acid (ABA), salicylic acid (SA), and methyl jasmonate (MeJA) stresses. The leaf samples treated with SA and MeJA were harvested at 0 h, 3 h, 12 h, and 24 h, while those treated with ABA were collected at 0 h, 3 h, 6 h, and 24 h. The expression level of glyceraldehyde-3-phosphate dehydrogenase (*GAPDH*) was used for data normalization. The bars represent the mean ± standard error (*n* = 3). Different lowercase letters on the top of the bars indicate the significant differences determined by Duncan’s new multiple range test (*p*-value < 0.05).

The qRT-PCR method was used to analyze the expression patterns of the *ShNPR1* gene under different exogenous stresses. The transcripts of *ShNPR1* were detected in the interaction between two different sugarcane varieties and *S. scitamineum* (Figure 9A). As compared to the control, the gene expression level of *ShNPR1* was significantly increased in the sugarcane smut-resistant variety YT96-86 at 3 days post-inoculation (dpi), but was significantly decreased in the sugarcane smut-susceptible variety ROC22 at 1 and 3 dpi. Under 100 μM ABA stress, there was a downregulation of *ShNPR1* at 3 and 24 h, whereas the highest expression was observed at 6 h, which was 2.09-fold higher than the control (Figure 9B). After 5 mM SA treatment, the gene expression level of *ShNPR1* was stabilized from 0 to 3 h, while its transcript abundance significantly reduced at 12 to 24 h (Figure 9C). Under 25 μM MeJA stress for 3 h, the gene expression level of *ShNPR1* was 2.16-fold higher than that of the control, following which it dropped to the control level at 12 and 24 h (Figure 9D).

### 2.11. Transient Expression of ShNPR1 in Nicotiana benthamiana Induced Plant Immune Response

**Figure 10:**
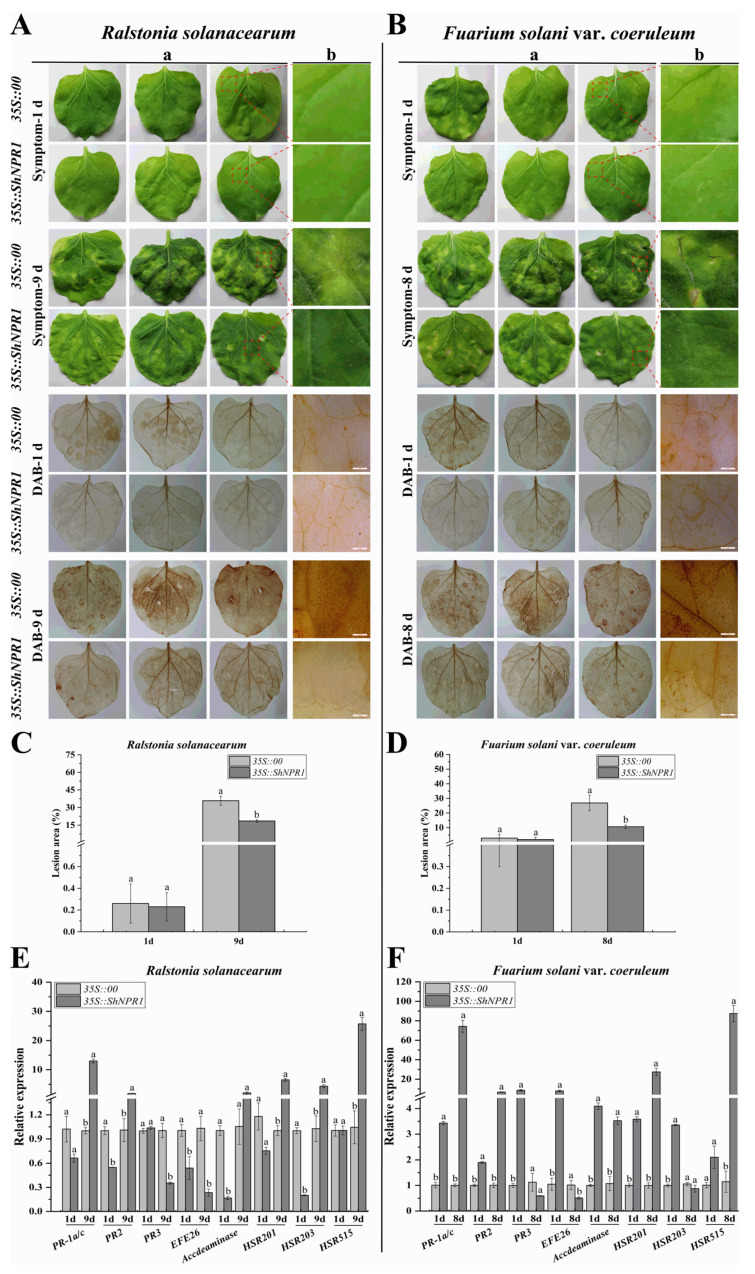
The immune effect of transient overexpression of *ShNPR1* in *Nicotiana benthamiana* after inoculation with *Ralstonia solanacearum* and *Fuarium solani* var. *coeruleum*. (**A**) The disease symptoms and 3,3-diaminobenzidine (DAB) staining of *N. benthamiana* leaves at 1 day and 9 days post-inoculation with *R. solanacearum*. (**B**) The disease symptoms and DAB staining of *N*. *benthamiana* leaves at 1 day and 8 days post-inoculation with *F*. *solani* var. *coeruleum*. (**C**) Lesion area of leaves after infection with *R. solanacearum*. (**D**) Lesion area of leaves after infection with *F*. *solani* var. *coeruleum*. (**E**) Expression of eight tobacco-immune-related marker genes in the *N*. *benthamiana* leaves 1 day and 9 days after inoculation with *R*. *solanacearum*. (**F**) Expression of eight tobacco-immune-related marker genes in the *N*. *benthamiana* leaves 1 day and 8 days after inoculation with *F*. *solani* var. *coeruleum*. The tobacco-immune-related marker genes, namely salicylic-acid-pathway-related genes *NtPR-1a*/*c*, *NtPR2*, and *NtPR3*; ethylene synthesis-dependent genes *NtEFE26* and *NtAccdeaminase*; hypersensitive reaction marker genes *NtHSR201*, *NtHSR203*, and *NtHSR515*. *NtEF-1α* was used for data normalization. (a) and (b) represent images taken with the Canon camera and a microscope (Bar = 2 mm), respectively. *35S::00*, the *Agrobacterium tumefaciens* strain carried the empty vector pEarleyGate 203; *35S::ShNPR1*, the *A*. *tumefaciens* strain carried the recombinant vector pEarleyGate 203-*ShNPR1*. The bars represent the mean ± standard error (*n* = 3). Different lowercase letters on the top of the bars indicate the significant differences determined by Duncan’s new multiple range test (*p-*value < 0.05).

The overexpression plasmid pEarleyGate 203 (*35S::00*) and the recombinant plasmid pEarleyGate 203-*ShNPR1* (*35S::ShNPR1*) carried by *A*. *tumefaciens* were transiently overexpressed in the leaves of *N*. *benthamiana*. The immune effect induced by transient overexpression of *ShNPR1* was analyzed by phenotypic observation, lesion area statistics, 3,3-diaminobenzidine (DAB) staining, and expression level analysis of immune-related marker genes in *N*. *benthamiana* (Figure 10).

There was no obvious difference in the symptom and DAB staining in *35S::00* and *35S::ShNPR1* leaves infected by *R. solanacearum* and *F. solani* var. *coeruleum* on one day. However, *35S::00* and *35S::ShNPR1* leaves that had been infected with *R. solanacearum* for nine days curled, wilted, and displayed white disease symptoms. Compared to *35S::00* leaves, this phenotype was weaker in *35S::ShNPR1* leaves (Figure 10A). Similarly, eight days after infection with *F. solani* var. *coeruleum*, leaves of *35S::00* with curled and wilted symptoms were more serious than that of *35S::ShNPR1* leaves (Figure 10B). After challenge by *F. solani* var. *coeruleum* and *R*. *solanacearum* for eight and nine days, the lesion areas of *35S::ShNPR1* leaves were all significantly lower than those of *35S::00* (Figure 10C,D). Furthermore, the DAB results demonstrated that the staining color in the *ShNPR1*-overexpressed tobacco leaves was lighter than that in the *35S::00* leaves after being infected by *R*. *solanacearum* for nine days and *F. solani* var. *coeruleum* for eight days (Figure 10A,B).

At one-day after post inoculation with *R*. *solanacearum*, the qRT-PCR analysis indicated that the expression level of four immune-related genes in *35S::ShNPR1* leaves, namely the SA-pathway-related gene *NtPR2*, ethylene (ET) synthesis-dependent genes *NtEFE26* and *NtAccdeaminase*, and hypersensitive response (HR) marker gene *NtHSR203*, was significantly lower than that in the control group (Figure 10E). However, on nine days of inoculation with *R*. *solanacearum*, the expression level of SA-pathway-related gene *NtPR-1a/c* and HR marker genes *NtHSR201* and *NtHSR515* was significantly higher than that in the control group by 13.00-, 6.53-, and 24.61-fold, respectively (Figure 10E). Furthermore, the expression level of the seven immune-related genes (*NtPR-1a/c*, *NtPR2*, *NtPR3*, *NtEFE26*, *NtAccdeaminase*, *NtHSR201*, and *NtHSR203*) in the *35S::ShNPR1* leaves was increased at one day after inoculation with *F*. *solani* var. *coeruleum*. Their expression level in the *35S::ShNPR1* leaves was 3.39-, 1.89-, 8.56-, 7.55-, 4.09-, 3.57-, and 3.35-fold that observed in the control, respectively (Figure 10F). Eight days after inoculation with *F*. *solani* var. *coeruleum*, the expression level of *NtPR-1a/c*, *NtPR2*, *NtAccdeaminase*, *NtHSR201*, and *NtHSR515* was all increased, and was 74.01-, 6.65-, 3.29-, 27.42-, and 76.38-fold that of the control, respectively (Figure 10F).

Based on these above results, overexpression of *ShNPR1* in *N. benthamiana* leaves enhances resistance to *R. solanacearum* and *F. solani* var. *coeruleum* infections, which was accompanied by the changing expression level of immune-related genes.

## 3. Discussion

Previous studies have shown that the *NPR1*-like genes not only take part in regulating plant growth and organ development, but also play vital roles in plant defense signal transduction pathways [12,15]. However, no systematic study on the *NPR1*-like genes has been conducted in *Saccharum*. In this study, the characteristics of the *NPR1*-like gene family in sugarcane and the regulatory role of *ShNPR1* in response to pathogen stress were analyzed, which should set a foundation for further functional identification of the *NPR1*-like genes in *Saccharum*.

In this study, 18 *NPR1*-like homologous genes with *Arabidopsis AtNPRs* were identified from the genome of *S. spontaneum* and were unevenly distributed (4, 6, and 8) among three clades (I, II, and III) in the phylogenetic tree (Figure 1). Moreover, three duplicate genes of *SsNPR1* were found in clade III, but none in clades I and II (Table S1). Due to the fact that five monocots (*Saccharum*, *O. sativa*, *Sorghum bicolor*, *T. aestivum*, and *Zea mays*) and five dicots (*A. thaliana*, *Carica papaya*, *Malus domestica*, *Persea Americana*, and *Populus trichocarpa*) have at least one member of the NPR1-like protein in each of the three clades (Table S3), the ancient duplication events resulting in functional divergence of *NPR1*-like genes may occur prior to the monocot-dicot split. Furthermore, SsNPR members in the same clade shared similar gene structures, conserved domains, and motifs presented in the *Arabidopsis NPR1*-like sequences (Figure 2), suggesting that their orthologs probably display similar biological functions in *Saccharum*. These findings indicate that the *NPR1*-like genes are highly conserved in numerous plant species.

WGDs or polyploidy was an important driving force in the evolution of organisms [32]. After WGDs, a proper balance in signaling and regulatory networks is maintained, while other types of duplication events (e.g., local, tandem, segmental, and aneuploidy) leave genes out of balance to varying degrees [33]. In this study, single copy, dispersed duplication, and WGD were all found in the *NPR1*-like gene family, and the expansion of the *SsNPR* gene family occurred mainly through WGD (Table S6). Synteny analysis revealed that the *NPR1*-like gene family in *S. spontanum* has experienced at least two rounds of WGDs (Figure 3), and the gene duplication events of *NPR1*-like genes in the monocots and dicots occurred after the divergence of monocots and dicots (Figure 1). Here, 13 out of 14 pairs of *SsNPR* duplicated genes have a Ka/Ks < 1 (Table S5), indicating that the replicated *NPR1*-like genes may be subject to strong selection pressure for purification.

Promoter *cis-*elements are essential for gene regulation [34]. MeJA and ABA both are important regulators in developmental processes and plant defense responses [35,36]. Previous studies showed that a similar antagonistic relationship exists between ABA and NPR1, e.g., ABA can inhibit the expression of the *NPR1* gene [37,38]. In our study, there were many stress-related and phytohormone-responsive *cis*-elements in the *SsNPR* promoters (Figure 4 and Table S8). Interestingly, each of the *NPR* promoters in *Saccharum* contained an ABRE *cis*-element, which is involved in ABA responsiveness (Figure 4). This suggests that the ABRE element may be involved in the regulation of *NPR1* by ABA. Furthermore, more than 88% of the promoter regions in the *NPR1*-like genes contained MeJA-responsive elements (CGTCA-motif and TGACG-motif) (Figure 4B), suggesting that *NPR1*-like genes may also involve a series of sugarcane physiological responses mediated by MeJA.

The nuclear localization signal in *NPR1*-like genes is closely related to its ability to timely and effectively induce the expression of defense genes [39]. *AtNPR1^C82A^* and *AtNPR1^C216A^* mutants cause NPR1 to depolymerize into monomers, localize in the nucleus, and stimulate the expression of defense genes in *Arabidopsis* [19]. Amino acid alignment analysis and subcellular location results showed that the ShNPR1 protein contained these two functional sites (Figure 6) and was localized in the nucleus (Figure 7). SA production results in an increase in the thioredoxins leading to the reduction of C156 and disassembly of the NPR1 oligomer [17,19]. Monomeric NPR1 is then translocated to the nucleus via a bipartite NLS where it induces the expression of *PR1* [40,41]. C156 is a highly conserved cysteines residue in NPR proteins (Figure 6), indicating that C156 may be involved in the formation of the NPR oligomer to regulate the expression of the *PR* gene.

The expression abundance of the *NPR1*-like gene in different plant tissues is not consistent [13,42]. There are 19 *BjuNPR* genes detected in the roots, stems, leaf, pod, and flowers of *B. juncea* var. *tumida* [42]. Different *BjuNPR* genes expressed in different tissues of *B. juncea* var. *tumida*, for example, *BjuNPR4-B,* exhibited a specific high expression in all tissues, while *BjuNPR6-A* was not expressed in the root [42]. Robert et al. [13] found that the expression of *Persea americana PaNPR* was higher in mature tissues than that in younger ones. In this study, *ShNPR1* expressed constitutively in the sugarcane tissues, with the highest expression level in the leaf and the lowest level in the bud (Figure 8). Interestingly, the tissue-specific expression patterns of sugarcane *NPR1*-like genes in different evolutionary branches were different (Figure 5A). These results indicate that *NPR1*-like genes may play different roles in the regulation of plant growth and development.

Previous research has shown that *NPR1*-like genes can be regulated by biotic stress [20,43,44]. Molla et al. [20] found that overexpressing *AtNPR1* in rice causes an enhancement in tolerance to sheath blight disease. Overexpression of the *MpNPR1* gene enhanced the resistance of *Malus pumila* to fire injury and fungal disease, and activated the expression of a series of downstream disease-resistance-related genes [43]. The *Gossypium hirsutum GhNPR1* gene was induced by MeJA, SA, and ET, and played a defensive role in *Fusarium oxysporum* and *Xanthomonas campestris* infections [44]. Here, after inoculation with the smut pathogen, the *ShNPR1* transcript abundance significantly increased in the smut-resistant variety YT96-86, but was the opposite in the smut-susceptible variety ROC22 (Figure 9A), suggesting that *ShNPR1* may be a positive responsive component of smut resistance in sugarcane. Phytohormones, such as SA, MeJA, ET, and ABA, play an essential role in the regulation of plant immune responses to pathogens [45]. *NPR1* is not only a receptor of SA but also a regulator of SAR, which plays a vital role in SA/JA signaling crosstalk [46]. In addition, SA-mediated redox modulation also plays an important role in the SA-mediated attenuation of the JA signaling pathway [47]. In this study, we found that SA reduced transcript levels of *ShNPR1* in *Saccharum* (Figure 3A), which was contrary to what was observed in *Persea americana* [13]. This result seems to be host-specific. On the other hand, after exogenous treatment with JA, the expression of *NPR1* increased first and then decreased, and similar results were also observed in banana plants [48]; however, a contradictory result was seen in *Brassica juncea* [49]. This difference in *NPR1* expression patterns may highlight the response diversity in monocotyledonous and dicotyledonous.

Yu et al. [25] found that overexpression of the *Secale cereale NPR1* gene could improve fusarium head blight resistance in wheat. Ramineni et al. [50] reported that the expression level of the *PR1* gene in the *BjNPR1*-overexpressing transgenic plants was significantly higher than that in the untransformed plants after being infected by *Sclerospora graminicola*. The hybrid lily ‘Sorbonne’ *LhSorNPR1* positively regulated the defense response of *Arabidopsis* to *P*. *syringae* pv. tomato DC3000 by elevating the transcript levels of SA-associated genes (*PR1*, *PR2*, and *PR5*) [51]. The overexpression of *BjNPR1* showed enhanced resistance to *Alternaria brassicae* and *Erysiphe cruciferarum* as there was a delay in symptoms and a reduced disease severity [49]. Similarly, in our study, the control leaves post-inoculated with *R. solanacearum* or *F. solani* var. *coeruleum* exhibited more severe disease symptoms than the leaves that overexpressed *ShNPR1* (Figure 10A–D). In addition, the transcript abundance of the tested tobacco-immune-related marker genes, especially SA-pathway-related genes and HR marker genes, was higher in the *35S::ShNPR1* leaves than that in the control (Figure 10E,F). These results reveal that the *ShNPR1* gene plays a positive role in plant defense responses, and the overexpression of *ShNPR1* in *N. benthamiana* leaves enhances resistance to *R. solanacearum* and *F. solani* var. *coeruleum* infections.

In summary, the *ShNPR1* gene may be involved in plant SA, JA, and HR signal regulatory networks and its expression can be induced by exogenous hormones ABA, SA, and MeJA. Although it is obvious that *ShNPR1* acts as an essential part of the biotic signaling pathway, further studies should be conducted to verify the function of the *ShNPR1* gene in stable transgenic plants.

## 4. Materials and Methods

### 4.1. Plant Materials and Treatments

Two different sugarcane genotypes, including smut-resistant variety YT96-86 and smut-susceptible variety ROC22, were provided by the Key Laboratory of Sugarcane Biology and Genetics and Breeding, Ministry of Agriculture and Rural Affairs (Fuzhou, China). These two materials were used to detect the expression level of *ShNPR1* in sugarcane infected with the smut pathogen. Ten-month-old sugarcane stalks were selected from the two varieties and cultured at 32 °C. After the buds germinated to 1–2 cm in length, an acupuncture inoculation method with 5 × 10^6^ mL^−1^ (containing 0.01% Tween-20, *v*/*v*) of smut pathogen suspension was used to inoculate the sugarcane buds, and the control group was inoculated with sterile water (containing 0.01% Tween-20, *v*/*v*) [52]. All inoculated materials were grown at 28 °C with a photoperiod of 16 h light and 8 h darkness. Five buds at time points of 0, 1, and 3 dpi were harvested and immediately frozen in liquid nitrogen, and stored at −80 °C until use.

For the analysis of the expression patterns of *ShNPR1* under different exogenous stresses, four-month-old ROC22 tissue culture seedlings were cultivated in water for about 10 d. The leaves of the seedlings were sprayed with 100 µM ABA, 5 mM SA (containing 0.01% Tween-20, *v*/*v*), and 25 µM MeJA (containing 0.1% ethanol and 0.05% Tween-20, *v*/*v*), respectively [53,54]. The leaf samples treated with SA and MeJA were harvested at 0, 3, 12, and 24 h, while those treated with ABA were collected at 0, 3, 6, and 12 h. As for the different sugarcane tissues, the root, bud, +1 leaf, stem epidermis, and stem pith were sampled from nine healthy and mature 10-month-old ROC22 plants [54]. All samples were immediately frozen in liquid nitrogen after being harvested and stored at −80 °C.

### 4.2. Identification and Characterization of the NPR1-like Gene Family

In order to identify the *NPR1-like gene* family in *Saccharum*, the genome of sugarcane ancestor *S. spontaneum* AP85-441 (http://www.life.illinois.edu/ming/downloads/Spontaneum_genome/) (accessed on 10 June 2021) [30] was selected. The hidden Markov model profile of BTB.hmm (Pfam: PF00651) was obtained from the Pfam database (http://pfam.xfam.org/) (accessed on 13 June 2021) [55]. The putative NPR1-like proteins identified by HMMER v3 software [56] were submitted to the CDD database (https://www.ncbi.nlm.nih.gov/cdd) (accessed on 13 June 2021) and BLAST program (https://blast.ncbi.nlm.nih.gov/Blast.cgi) (accessed on 13 June 2021) to check for the N-terminal BTB/POZ domain and ANK repeats [14]. The basic properties including MW, *p*I, the grand average of hydropathicity (GRAVY), and instability index were analyzed using the online ExPASy-ProtParam tool (http://web.expasy.org/protparam/) (accessed on 2 July 2021). The subcellular localization and secondary structure of NPR1-like proteins were predicted using Plant-mPloc (http://www.csbio.sjtu.edu.cn/bioinf/plant-multi/) (accessed on 5 July 2021) and Prabi (https://npsa-prabi.ibcp.fr/cgi-bin/npsa_automat.pl?page=/NPSA/npsa_server.html) (accessed on 10 July 2021), respectively.

### 4.3. Analysis of Phylogenetic, Gene Structure, Motif Composition, Gene Duplication, and Synteny

The NPR1-related protein sequences of *O. sativa* (Os), *Z. mays* (Zm), *S. bicolor* (Sb), *Musa acuminate* (M), *T. aestivum* (Ta), *A. thaliana* (At), *C. papaya* (Cp), *Cocos nucifera* (Cn), *Morus multicaulis* (Mu), *Glycine max* (Gm), *M. domestica* (Mp), *Vitis vinifera* (Vv), *P. trichocarpa* (Pt), and *P. americana* (Pa) were downloaded from the GenBank database (https://www.ncbi.nlm.nih.gov/genbank/) (accessed on 5 October 2021) (Table S3). The full-length proteins from *Saccharum* and 14 other plant species were aligned by MUSCLE within MEGA X [57]. The phylogenetic tree was constructed by MEGA X with the neighbor-joining (NJ) method and the parameters of complete deletion, Poisson correction, and 1000 bootstrap replicates [57]. Then, the phylogenetic tree was displayed by ITOL v6 (https://itol.embl.de/) (accessed on 10 October 2021).

The Multiple Expectation Maximization for Motif Elicitation online program (http://meme-suite.org/tools/meme) (accessed on 18 October 2021) was applied to identify the conserved motifs of NPR1-like proteins [58]. Gene structures of the identified *SsNPR1-*like gene IDs were extracted from the GFF3 file in the genome data. TBtools was used to display the phylogenetic tree, conserved motifs, and gene structures [59]. The Multiple Collinearity Scan toolkit (MCScanX) was used to analyze the duplication pattern and synteny of the *NPR1*-like genes [60]. TBtools was used to calculate the values of Ka/Ks between orthologous gene pairs [59].

### 4.4. Cis-Acting Regulatory Elements Analysis in the Promoter Regions

The promoter regions, 2 kb of the genomic DNA sequence upstream of the translation start site of each sugarcane *NPR1-*like gene, were identified by searching the genome of *S. spontaneum.* These promoter sequences were then submitted to the PlantCARE online program (https://bioinformatics.psb.ugent.be/webtools/plantcare/html/) (accessed on 25 October 2021) to identify *cis*-regulatory elements [61]. Then, the results were visualized using TBtools [59].

### 4.5. Expression Profiles of Sugarcane NPR1-like Genes Based on the Available Transcriptome Datasets

The bud, stem pith, epidermis, leaf, and root of 10-month-old ROC22 sugarcane plants were collected and frozen in liquid nitrogen. The experiment has three biological replicates, and each biological replicate contains three samples. According to the transcriptome data reported by Que et al. [62], the buds of ROC22 (sugarcane smut-susceptible variety) and *Saccharum* spp. hybrid YC05–179 (sugarcane smut-resistant variety) were taken after being infected with *S. scitamineum* for 0, 1, 2, and 5 days. Based on the reference genome of *S. spontaneum* [30], the above RNA samples were sent to BMK Biotechnology Co, Ltd. (Beijing, China) for RNA-seq analysis. The resulting raw data were treated by FASTP and HISAT2 programs, respectively, to improve the sequence quality and map the sequence data to the reference genome. The expression profiles of *NPR1-*like genes in different sugarcane tissues and under *S. scitamineum* stress were transformed by log_2_ FPKM (the number of fragments per kilobase of transcript per million mapped in the transcriptome data). The heat map was constructed with TBtools [59].

### 4.6. Gene Cloning and Bioinformatic Analysis of ShNPR1

Using the first-strand cDNA of ROC22 leaves as a template and the *ShNPR1*-cDNA (Table S10) as a primer, the cDNA sequence of *ShNPR1* was cloned. The PCR program temperature conditions were 94 °C for 5 min, followed by 35 cycles of 94 °C for 30 s, 54 °C for 45 s, and 72 °C for 2 min, and an elongation step at 72 °C for 10 min. The PCR product was constructed into the cloning vector Blunt-Zero (Transgen Biotech, China, Beijing) and transformed into *Escherichia coli* DH5α. The positive recombinant vector Blunt-Zero-*ShNPR1* was obtained after sequencing (Fuzhou Shangya Biotechnology Co., Ltd., Fuzhou, China). ORF Finder (https://www.ncbi.nlm.nih.gov/orffinder/) (accessed on 12 November 2021) and the conserved domains database (CDD) (http://www.ncbi.nlm.nih.gov/Structure/cdd/wrpsb.cgi) (accessed on 13 November 2021) were used to predict the ORF and the conserved domain of the *ShNPR1* gene. The sequences were aligned using the Clustal Omega program (https://www.ebi.ac.uk/Tools/msa/clustalo/) (accessed on 5 March 2022) and visualized in Jalview Version 2.11.2.2. DNAMAN 6.0 software was used to perform sequence homology analysis.

### 4.7. Subcellular Localization Analysis of ShNPR1

The primer *ShNPR1*-gate (Table S10) was used to amplify the *ShNPR1* ORF (without the stop codon). This fragment was then ligated to the subcellular localization vector pFAST-R05 by using gateway technology. After sequencing verification, a positive recombinant plasmid pFAST-R05-*ShNPR1* was obtained. To confirm the subcellular location of the ShNPR1 protein, the *A. tumefaciens* GV3101 cells containing the pFAST-R05-*ShNPR1*-*GFP* vector or pFAST-R05-*GFP* vector were centrifuged and resuspended by the induction medium (10 mM MES, 10 mM magnesium chloride (MgCl_2_), and 200 μM acetosyringone, pH 5.0–5.4) at an OD_600_ of 0.8 [63]. Then, they were injected into *N*. *benthamiana* leaves. The infected plants were cultured for 2 d at 28 °C (16 h of light and 8 h of darkness). In addition, 10 μg/mL of 4′, 6-diamidino-2-phenylindole (DAPI) was used for nuclear staining. Subcellular localization results were observed under laser scanning confocal microscopy (Leica TCS SP8, Wetzlar, Germany) using a 10× lens, a 488 nm green fluorescence excitation wavelength, and a 458 nm chromatin DAPI filter.

### 4.8. Quantifification of ShNPR1 Expression by qRT-PCR Analysis

On the NCBI online software, the specific qRT-PCR primers ShNPR1-QF/R (Table S10) were designed based on the *ShNPR1* gene sequence. The glyceraldehyde-3-phosphate dehydrogenase (*GAPDH*) gene was used as an internal reference [64]. The 10-fold diluent cDNAs of different sugarcane tissues (root, bud, leaf, stem pith, and stem epidermis) and samples treated with ABA, SA, and MeJA were used as the qRT-PCR templates. RNA extraction with Trizol^®^ Reagent (Invitrogen, Carlsbad, CA, USA) and qRT-PCR analysis with ChamQ™ Universal SYBR@ qPCR Master Mix were referenced to our previous study [65]. The 20 μL reaction system of qRT-PCR contained 10 μL of SYBR Green Master Mix, 0.4 μL of 10 μM forward primer, 0.4 μL of 10 μM reverse primer, 1.0 μL of 10× diluted cDNA template, and 8.2 μL of sterile distilled water. The qRT-PCR procedure was 50 °C for 2 min, 95 °C for 10 min, and 40 cycles of 95 °C for 15 s and 60 °C for 1 min. After that, the melting curves were analyzed. The 2^−ΔΔCt^ method was used to calculate the qRT-PCR data [66]. The significance level of the data was analyzed using DPS 9.50 software, and graphs were plotted using Origin 8.0 software. All these data points were mean ± standard error (*n* = 3). Bars superscripted by different lowercase letters indicate significant differences, as determined by Duncan’s new multiple range test (*p*-value < 0.05).

### 4.9. Transient Expression of ShNPR1 in Nicotiana benthamiana

The plant overexpression vector pEarleyGate 203-*ShNPR1* was constructed using the gateway technology. The empty vector pEarleyGate 203 and the recombinant vector pEarleyGate 203-*ShNPR1* carried by *A. tumefaciens* GV3101 were transiently overexpressed in the leaves of four-week-old *N. benthamiana* [67]. The one-day overexpression *N. benthamiana* leaves were inoculated with tobacco *R*. *solanacearum* and *F*. *solani* var. *coeruleum* according to the method of Ren et al. [65]. All materials were kept at 28 °C for 16 h of light and 8 h of darkness, and phenotypic alterations were noted. The Adobe Photoshop CS5 software was used to analyze the leaf lesion area. In addition, the leaves inoculated with *R*. *solanacearum* for 1 d and 9 d, or inoculated with *F*. *solani* var. *coeruleum* for 1 d and 8 d were collected for DAB histochemical detection to test hydrogen peroxide (H_2_O_2_) accumulation and qRT-PCR analysis of the expression level of the tobacco-immune-related marker genes. These tobacco-immune-related marker genes included the HR marker genes *NtHSR201*, *NtHSR203*, and *NtHSR515* [68]; SA-related genes *NtPR-1a/c*, *NtPR2,* and *NtPR3* [69]; ET synthesis-dependent genes *NtEFE26* and *NtAccdeaminase* [70] (Table S10). *NtEF-1α* was used as an internal reference gene (Table S10).

## Data Availability

Not applicable.

## Supplementary Materials

The following supporting information can be downloaded at: https://www.mdpi.com/article/10.3390/ijms23147984/s1.
