# Supplementary material for: Characterization and Functional Implications of the Nonexpressor of Pathogenesis-Related Genes 1 (NPR1) in Saccharum
Source: Int J Mol Sci. 2022 Jul 20;23(14):7984. doi: 10.3390/ijms23147984 (PMC9317693; doi:10.3390/ijms23147984)
Supplement: Supplementary file 1 [file ijms-23-07984-s001.zip › supplement figures.pdf]

Characterization and Functional Implications of the NONEXPRESSOR OF PATHOGENESIS-RELATED GENES 1 (*NPR1*) in *Saccharum*

Shoujian Zang [1], Liqian Qin [1], Zhennan Zhao [1], Jing Zhang [1], Wenhui Zou [1], Dongjiao Wang [1], Aoyin Feng [1], Shaolin Yang [1,2], Youxiong Que [1,3,*] and Yachun Su [1,3,*]

1 Key Laboratory of Sugarcane Biology and Genetic Breeding, Ministry of Agriculture and Rural Affairs, Fujian Agriculture and Forestry University, Fuzhou 350002, China; zangshoujian2020@163.com (S.Z.); qinliqian0000@163.com (L.Q.); zn2008@139.com (Z.Z.); zhashoveljing@163.com (J.Z.); zwh19961546644@126.com (W.Z.); dongjiaow@126.com (D.W.); feng_aoyin98@163.com.(A.F.)

2 Yunnan Key Laboratory of Sugarcane Genetic Improvement, Sugarcane Research Institute, Yunnan Academy of Agricultural Sciences, Kaiyuan 661600, Yunnan, China; cbttxsysl@foxmail.com (S.Y.)

3 Key Laboratory of Genetics, Breeding and Multiple Utilization of Crops, Ministry of Education, College of Agriculture, Fujian Agriculture and Forestry University, Fuzhou 350002, Fujian, China

* Correspondence: queyouxiong@126.com (Y.Q.); syc2009mail@163.com (Y.S.); Tel.: +86-591-8385-2547 (Y.Q. & Y.S.)

```
    ATGGAGCCGTCGTCGTCCATCACGTTTGCGTCGTCGTCGTCGTACCTGTCCAACGGCTCCAGCCCCTGTTCCGTCGCGATGCCGCCGCCG
1   M E P S S S I T F A S S S S Y L S N G S S P C S V A M P P P
    GGGCCGCCCCAGGCTCCTCCCTTGGCTGCCGGCGAGTTATGGGGTGGTGGAGTCGCTGCCGCAGGGAGCGGAAGCAGCGTGGAGGCGGTG
31  G P P Q A P P L A A G E L W G G G V A A A G S G S S V E A V
    AGCCTGAACCGCCTCAGCAAGAACCTCGAGCGGCTGCTCCTCGACCCGGACCTAGACTGCAGCGACGCCGACGTCGAGGTGCCCGACGGC
61  S L N R L S K N L E R L L L D P D L D C S D A D V E V P D G
    GGGCCGCCCGTACCCATCCACCGCTGCATACTTGCCGCGCGCAGCGACTTCTTCTACGACCTCTTCGCCGCGCGCGGCCGCGGTGGGGCA
91  G P P V P I H R C I L A A R S D F F Y D L F A A R G R G G A
    GCGCGCGGTGATGCGACCGCCGGCGCCGGAGGAGCAGCGGAGGGGGCTGCCAGTGGAAGGCCGCGGTACAAGATGGAGGAGCTCGTCCCC
121 A R G D A T A G A G G A A E G A A S G R P R Y K M E E L V P
    GGGGGTCGCGTGGGGCGCGAGGCGTTCCAGGCGTTTCTGGGGTACATGTACACCGGCAAGCTCCGGCCGTCACCGGTCGACGTGGTCTCT
151 G G R V G R E A F Q A F L G Y M Y T G K L R P S P V D V V S
    TGTGCTGACCCAGTGTGCCCTCACGATTCGTGCCCGCCGGCCATCAGGTCGGCGGTCGAGCTCATGTACGCGGCGTGTACCTTCAAGATC
181 C A D P V C P H D S C P P A I R S A V E L M Y A A C T F K I
    CCCGAGCTCACCTCGCTCTTCCAGCGGCGGCTTCTTAACTTTGTAGACAAGACTCTAGTGGAGGATGTCATTCCTATTTTGAAAGTTGCT
211 P E L T S L F Q R R L L N F V D K T L V E D V I P I L K V A
    TCCCACTCAGGGCTGACTCAAGTGATTGACAAATGTATTCAAAGGATTGCTAGATCAGATCTTGACGATATATCTTTGGATAAGGAGCTC
241 S H S G L T Q V I D K C I Q R I A R S D L D D I S L D K E L
    CCTCCAGAAGCTGTTGAGGAGATAAAAAATTTGCGCAAGAAGTCACCAACTGCTGATGGTGATGGCGATGCGTTCATTTCGGACCCTGTG
271 P P E A V E E I K N L R K K S P T A D G D G D A F I S D P V
    CATGAGAAAAGAGTCAGAAGAATCCACAGGGCACTCGACTCTGATGATGTTGAGCTTGTTAAGTTGCTTCTGAATGAGTCTGACATCACA
301 H E K R V R R I H R A L D S D D V E L V K L L L N E S D I T
    TTAGATGATGCCAATGCATTACACTATGCTGCTTCTTACTGTGATTCTAAAGTTGTCTCAGAGCTATTAGATTTGGCGCTGGCTAACTTA
331 L D D A N A L H Y A A S Y C D S K V V S E L L D L A L A N L
    AATTTGAAGAATAGCCGTGGATACACAGCACTCCACTTGGCTGCTATGAGGAGAGAACCAGCTATCATTATGTGTCTCCTTAACAAAGGG
361 N L K N S R G Y T A L H L A A M R R E P A I I M C L L N K G
    GCAAATGTATCACAACTGACAGCTGATGGCAGGAGCGCAATTGGTATTTGTCGGAGGTTAACAAGATTAAAAGACTACAATACAAAGATG
391 A N V S Q L T A D G R S A I G I C R R L T R L K D Y N T K M
    GAGCAGGGTCAAGAATCAAATAAAGATAGGCTGTGTATAGATATTCTAGAGAGGGAGATGATGCGAAATCCTATGGCGGTGGAAGATGCT
421 E Q G Q E S N K D R L C I D I L E R E M M R N P M A V E D A
    GTCACCTCGCCTTTATTGGCAGATGATCTTCACATGAAGCTTCTCTACCTGGAAAACAGAGTTGCATTTGCTAGATTGTTCTTTCCTGCT
451 V T S P L L A D D L H M K L L Y L E N R V A F A R L F F P A
    GAAGCCAAGGTCGCCATGCAAATTGCACAAGCAGACACCACAGAAGAATTCGGCGGTATAGTTGCAGCAAGTACTTGTGGTAAACTGAGG
481 E A K V A M Q I A Q A D T T E E F G G I V A A S T C G K L R
    GAGGTCGACCTTAATGAGACGCCAGTCACACAAAACAAAAGACTCCGTTCAAGGGTAGATGCACTGATGAAAACAGTGGAGCTGGGCCGT
511 E V D L N E T P V T Q N K R L R S R V D A L M K T V E L G R
    CGGTACTTCCCGAACTGCTCGCAGGTGCTGGACAAATTCCTGGAGGACGACCTGCCGGACGGGCTGGACCAGTTCTACCTCCAGAGGGGC
541 R Y F P N C S Q V L D K F L E D D L P D G L D Q F Y L Q R G
    ACCGCCGACGAGCAGAAGGTGAAGAGGATGCGCTTCTGTGAGCTGAAGGAGGACGTGCTGAAGGCCTTTAGCAAGGACAAGGCGGACAGC
571 T A D E Q K V K R M R F C E L K E D V L K A F S K D K A D S
    AGCATGTTCTCGGGCCTGTCTTCATCGTCGTCGTGCTCGCCACCCCAGAAATCCACCAAGAGGTGA
601 S M F S G L S S S S S C S P P Q K S T K R *
```

Figure. S1 Nucleic acid sequence and the coding amino acid sequence of *ShNPR1* gene. The red font was the cysteine site; the red box, green box and orange box sequences represented the BTB/POZ conservative domain, ankyrin repeats and NPR1-like C-terminal region, respectively. The purple sequence was the nuclear location signal, and * represented stop codon.
